# Energy expenditure and effort of patients with stroke during sit to stand: A pilot study

**DOI:** 10.4102/sajp.v80i1.2022

**Published:** 2024-05-10

**Authors:** Tracy Harington, Nicolette Comley-White, Ronel Roos

**Affiliations:** 1 Department of Physiotherapy, Faculty of Health Sciences, University of the Witwatersrand, Johannesburg, South Africa

**Keywords:** energy expenditure, modified Borg scale, perceived effort, physiotherapy, sit to stand, stroke

## Abstract

**Background:**

Sit-to-stand (STS) is a mechanically demanding task. Little is known about the energy expenditure (EE) and the perceived effort of patients with stroke during STS.

**Objectives:**

The objectives of our study were to assess the perceived effort and EE of patients with stroke when moving from STS and to determine whether an association between actual energy expended and patient-perceived effort exists.

**Method:**

This descriptive cross-sectional pilot study assessed participants’ EE and perceived effort during STS, with a triaxial accelerometer and the modified Borg scale (MBS), respectively.

**Results:**

The team screened 428 individuals for potential inclusion, with nine participants (*n* = 5 female, 55.5%) meeting the criteria for our pilot study. Participants had a mean age of 52.77 (standard deviation [SD] ± 11.33) years, the majority had a haemorrhagic stroke (*n* = 6, 66.6%) and left hemiplegia (*n* = 6, 66.6%), and they were assessed 9.11 (SD ± 6.57) days post-stroke. The mean EE during STS was 2.82 (SD ± 1.9) kCal. Most participants (*n* = 7, 77.77%) perceived STS as more than a ‘moderate’ effort on the MBS. The correlation coefficient between the metabolic equivalent of task (METs) and MBS was *r* = 0.34 (*p* = 0.38).

**Conclusion:**

Our study found a fair positive correlation between METs and MBS for patients with stroke during STS.

**Clinical implications:**

The increased EE shown can be a key point for rehabilitation to lessen the extent of EE during STS. Further research is warranted.

## Introduction

Stroke and ischaemic heart disease are the leading ranked causes of disability-adjusted life-years in the 50–75 age group as per the 2019 Global Burden Disease Study (Murray et al. 2020). Nearly 5 million people die from stroke with 5 million people left permanently disabled (Gund et al. 2013).

Disability post-stroke is influenced by multiple factors: it differs according to the amount of recovery that occurs neurologically, where the lesion occurred, the premorbid status of the patient and the environmental support systems in place (Teasell & Hussein 2014). During hospitalisation, patients’ physical activity level is lower than their pre-admission level. In addition, little time is spent on moderate to high-level physical activity (PA) tasks during rehabilitation sessions (West & Bernhardt 2012).

One of the most frequent functional tasks performed daily is the movement of standing up from a seated position (Pollock et al. 2014). Evidence suggests that it may be one of the most mechanically demanding functional tasks that people perform on a regular basis (Pollock et al. 2014). The action of coming from sitting to standing (STS) is where the body’s centre of mass (CoM) moves upward from a seated position to a standing position without the loss of balance (Roebroeck et al. 1994). Being able to perform STS requires sufficient balance, adequate joint range of motion (ROM), coordination and muscle strength (Prudente, Rodrigues-De-Paula & Faria 2013). The ability to move the CoM of the body forward from a wide base of support to a narrow one is also needed (Prudente et al. 2013). It is an important precursor to walking and is essential for the prevention of falls and independent living (Culhane et al. 2005). For one to achieve STS, it is necessary for muscle activation in a coordinated manner, as a means of controlling segmental mobility and the total body momentum (Prudente et al. 2013).

Energy expenditure (EE) is the production of energy from the combustion of natural sources in the form of fat, alcohol, carbohydrate or protein (Hills, Mokhtar & Byrne 2014). During this process, oxygen is used and carbon dioxide is made. It is referred to as direct calorimetry where the measurement of EE involves the amount of heat loss directly or heat production (Hills et al. 2014). Training received, spasticity and degree of weakness in patients with stroke may cause a variation in the EE, which may lead to higher levels of energy expended to accomplish the task thus further potentially decreasing engagement in this task (Singh, Stewart & Franzsen 2011).

Post-stroke, patients’ problems relating to the ability to STS independently can be attributed to several factors. According to Jeyasurya et al. (2013), static and dynamic stability, extensor effort, momentum transfer energy and subjective preference can affect STS. Ratings of perceived exertion (RPE) are thought to be very important in the regulation of intensity during self-paced PA (Abbiss et al. 2015). The development of effort and exertion perception is an intricate process, which involves copious neural processes taking place in various regions within the brain (Abbiss et al. 2015).

The EE of adults with no known disease has previously been calculated (Boukadida et al. 2015), and the determinants of STS in individuals with hemiparesis post-stroke have also been established (Jeyasurya et al. 2013). The perceived effort has been established for patients with stroke during walking and other activities (Compagnat et al. 2017). However, the amount of energy expended and the perceived effort in a patient with stroke during STS is still unknown. A literature review carried out by Boukadida et al. (2015) concluded that there is a requirement for further research to enhance the understanding of STS activity and to outline the effect of clinical impairments related to stroke. The main goal would be to have a clearer understanding of STS and how it can be used to advance rehabilitation programmes post-stroke (Boukadida et al. 2015).

Our pilot study aimed to determine the EE of a patient with stroke during STS. There were three study objectives: (1) to establish EE during STS in patients with stroke, (2) to establish perceived effort during STS in patients with stroke and (3) to establish the association between actual energy expended and perceived effort during STS in patients with stroke.

## Research methods and design

Our descriptive cross-sectional pilot study took place at two large hospitals in Johannesburg, South Africa, and a sample of convenience was used.

Participants were included if they were admitted to the hospital because of a haemorrhagic or ischaemic stroke or attending outpatient physiotherapy because of stroke rehabilitation. Additionally, those aged between 35 years and 80 years, had a stroke in the last 3 months and presented with hemiparesis or hemiplegia and were able to demonstrate that they could perform STS without the use of either an assistive device or their upper limbs when standing up from a surface where their feet are flat on the ground and hips and knees flexed at 90°, were considered for inclusion. The participants were excluded if they had a previous stroke or were unable to follow instructions or provide feedback on perceived effort when using the modified Borg scale (MBS) because of any perceptual, language or cognitive deficits.

The sample size was calculated using the STATA programme with the test comparing one mean to a reference value, with power at 80% and α at 0.05. Two mean estimates: 0.019 (± 0.45) is the rate of 1/min-1 for the STS transition reported in a study by Júdice et al. (2016), as used during sample calculation. Forty-five patients with stroke were required for a full study, and nine participants were deemed feasible for our pilot study.

An RT3 triaxial accelerometer is a self-calibrating electronic device that is fitted over the participant’s hip and is linked to a computer using a body composition indicator docking station. During movement, the device accurately and objectively measures the EE of individuals (Mathie et al. 2004; Verbunt et al. 2001). It has been found to have a high degree of reliability during measurement with little variation over a period (Mathie et al. 2004) and has been shown as a reliable outcome measure in patients with stroke (Rand et al. 2009). Krasnoff et al. (2008) showed a minimal shaker variance with coefficients of variation (CVs < 0.52%). The study also revealed good reliability within the RT3s (CVs < 1.81%).

The MBS was used to assess participants’ perceived effort when performing STS. The MBS appears to be a reasonable indicator of the intensity of exercise after stroke when performed at moderate (60% – 70% Vo2peak) but not high-intensity exercise level (80% Vo2peak) (Sage et al. 2013). A study carried out by Grant et al. (1999) tested a group of active male volunteers to determine which of the subjective scales, the Likert Scale, the visual analogue scale or the MBS, were more sensitive to change and reproducible in the assessment of general fatigue and breathlessness during sub-maximal exercise. In general, the MBS proved to be the most sensitive in assessing change (Roos & Eales 2002). To determine participants’ severity post-stroke, the National Institutes of Health Stroke Scale (NIHSS) was used in our study. The NIHSS is most commonly used as a scoring system in stroke research trials; it is a valid, reproducible scale that measures neurological deficits for physical and cognitive functioning. The scale is scored between a range of 0 and 42, and the items are graded on a 3- or 4-point ordinal scale; 0 means no impairment and the higher the score the greater the severity of impairment. The severity of the stroke may be presumed based on the NIHSS scores as follows (Brott et al. 1989): (1) very severe: > 25, (2) severe: 15–24, (3) mild to moderately severe: 5–14 and (4) mild: 1–5. A calibrated scale was used to measure participants’ weight in kilograms (kg), and a stadiometer was used to measure participants’ height in centimetres (cm). Participants’ body mass index (BMI) was calculated from these measurements.

Patients who met the inclusion criteria were invited to be part of our study through an information letter, and once they signed the informed consent form, they were assessed. The demographic information, age, duration of stroke and the NIHSS were recorded by the principal investigator on the demographic questionnaire, along with their height, weight and BMI. To measure the EE, the participants were fitted with the RT3 on a Velcro belt, which was worn on the participant’s left hip at the midpoint of the iliac crest. Once activated, participants were required to perform the STS activity twice. It was measured twice to familiarise the participant with the activity and the second performance was used with data analysis. Each STS was timed with a stopwatch. The MBS was used on the completion of the STS task and findings were recorded on the demographic questionnaire.

The data were tested for normality in IBM SPSS version 28 using a Shapiro-Wilk test, and findings are presented as means (standard deviation [SD]) and medians (interquartile range [IQR]). The correlation was determined using the Pearson correlation test. The strength of association of the correlation coefficient between two variables was measured and interpreted as follows: 0.00–0.25 = little or no relationship, 0.25–0.50 = fair relationship, 0.50–0.70 = moderate to fair relationship and above 0.75 = good to excellent relationship (Portney & Watkins 2009). The demographics and clinical profile data were analysed in Excel™ using means and standard deviations and IBM SPSS version 28, and a statistician was consulted for the statistical analysis tests.

### Ethical considerations

Ethical clearance was received from the Human Research Ethics Committee (Medical) of the University of the Witwatersrand (Certificate number: M180209), and permission was obtained from the study sites and the National Health Research Database (NHRD). Participants consented in writing to participate in our study. All data were coded for anonymity and kept confidential.

## Results

The team screened 428 patients for potential inclusion in our study. Four hundred and nineteen patients were excluded because of having had a previous stroke (*n* = 118), age not aligning to inclusion criteria (*n* = 92), individuals not being able to perform STS as required (*n* = 94), being clinically unstable (*n* = 25) and 90 patients being cognitively impaired. Nine participants were included in our pilot study, of which the majority were female (*n* = 5; 55.5%), had a haemorrhagic stroke (*n* = 6; 66.6%) and presented with a left hemiplegia (i.e. right hemisphere stroke) (*n* = 6; 66.6%) (Table 1).

**TABLE 1:** Demographic and clinical profile of the study population (*N* = 9).

Variable	Category	*n*	%
Gender	Male	4	44.4
	Female	5	55.5
Stroke type	Ischaemic	3	33.3
	Haemorrhagic	6	66.6
Side of hemiplegia	Right	3	33.3
	Left	6	66.6

The mean (SD) age of the study participants was 52.77 (± 11.33) years, with stroke severity grading a median of 4 (IQR 3) with borderline BMI mean finding (Table 2).

**TABLE 2:** Age, days since the stroke, National Institutes of Health Stroke Scale stroke severity, height (m), weight (kg) and body mass index for the study population (*N* = 9).

Variable	Median	IQR	Mean	SD
Age (years)	-	-	52.77	11.33
Age, female, (*n* = 5)	-	-	54.4	9.56
Age, males, (*n* = 4)	-	-	50.75	14.52
Days since stroke	-	-	9.11	6.57
NIHSS†	4	3	4.55	1.94
Height, m	-	-	1.65	0.07
Weight, kg	-	-	66.00	12.73
BMI‡	-	-	24.31	5.36

Note:

† , Interpretation of NIHSS severity grading (/42): 0–5 mild, 6–14 mild to moderately severe, 15–24 severe and > 25 very severe;

‡ , BMI: < 18.5 underweight, 18.5–24.9 healthy weight, 25.0–29.9 overweight and >35 obese.

IQR, interquartile range; BMI, body mass index; kg, kilogram; m, metres; NIHSS, National Institute of Health Stroke Scale; SD, standard deviation.

The mean time taken for STS was 6.04 (±4.03) s (female = 6.08) (5.5 – 7); male = 3.4 (2.7 – 6.21) and the total caloric expenditure value for STS was 2.82 (±1.9) kCal. The males expended almost twice the energy (4.39 [IQR 2.99–6.05 kCal/min] during STS compared to that of the female participants (2.89 [1.4–2.10 kCal/min]) (Table 3).

**TABLE 3:** Caloric expenditure and metabolic equivalent of tasks during sit-to-stand for the study population (*N* = 9).

Variable	Mean	SD	Median	IQR
Time taken, s	6.04	4.03	-	-
Total caloric expenditure, kCal	2.82	1.90	-	-
Total METS, kCal/min	2.99	1.89	-	-
Females total METS, kCal/min	-	-	2.89	1.40–2.10
Males total METS, kCal/min	-	-	4.39	2.99–6.05

METs, metabolic equivalent of tasks; IQR, interquartile range; SD, standard deviation.

The three planes assessed showed that there was more movement into the vertical plane: 538 (425 – 785), followed by the mediolateral plane: 325 (220 – 638) and then the anteroposterior plane: 28 (3 – 456).

The majority of participants perceived STS as more than a ‘moderate’ effort rating (*n* = 7, 77.77%) (Table 4). The mean MBS rating of participants was 3.44 (±1.51) with male participants rating their effort higher (4 [IQR 2.5–5.5]) than female participants (3 [IQR 3–4]).

**TABLE 4:** The perceived effort to complete the sit-to-stand task in the study population (*N* = 9).

MBS rating	*n*	%
0	0	0.00
0.5	0	0.00
1	1	11.11
2	1	11.11
3	3	33.33
4	2	22.22
5	1	11.11
6	1	11.11
7	0	0.00
8	0	0.00
9	0	0.00
10	0	0.00

Note: 6 and 8 do not have ratings, 0 = nothing at all; 0.5 = very, very slight; 1 = very slight; 2 = slight; 3 = moderate; 4 = somewhat severe; 5 = severe; 7 = very severe; 9 = very, very severe; 10 = maximal.

MBS, modified Borg scale.

The correlation coefficient between the subjective perceived effort and physical effort as per EE was *r* = 0.61 (*p* = 0.08) and subjective perceived effort and metabolic equivalent of task (MET) level *r* = 0.34 (*p* = 0.38), but the findings were not statistically significant.

## Discussion

Our pilot study aimed to establish the feasibility of determining the EE of patients with stroke during STS. Physical effort evaluation and monitoring are important to highlight exercise intensity levels during the rehabilitation of patients following stroke.

The mean METs of the participants during STS in our study was 2.99 (± 1.89) kCal/min indicating that the intensity level of this functional task was of moderate-intensity level. This finding was much higher compared to literature related to EE during STS in healthy individuals. In a study carried out by Júdice et al. (2016), they reported an EE of a single STS in a normal healthy population to be 0.32 kCal/min. In another study, also carried out in a normal healthy population, results showed that the EE during STS was 0.22 (± 0.09) kCal/min; however, they only had 19 participants with a mean age of 23.1 (± 1.9) years compared to Júdice et al. (2016) who had 50 participants with a mean age for males 32.5 (± 11.4) years and females 38.0 (± 15.7) years (Hatamoto et al. 2016). This indicates that our study participants with stroke used almost eight times more energy than the unaffected test participants in the Júdice et al. (2016) study. To the authors’ knowledge, there are no other studies establishing the EE of patients with stroke during STS.

The time taken to complete STS: the mean time taken for the STS was 6.04 (± 4.03) s where the female’s median time was on the 75th percentile of the IQR with 6.08 (5.5 – 7) s and the males closer to the 25th percentile of the IQR at 3.4 (2.7 – 6.21) s. A study carried out with patients with stroke had an STS time of 3.57 (± 1.69) s (Arcelus et al. 2009) and a sample of adults with no known disease had an STS duration of 2.88 (± 1.13) s for older adults and 2.31 (± 0.63) s in the younger adults (Arcelus et al. 2009), whereas the patients with stroke had an STS duration of 3.57 s (± 1.69) (Arcelus et al. 2009). Cameron et al. (2003) assessed a group of 15 patients with stroke who required almost twice as much time to complete the STS (3.86 [± 1.52] s) compared to a control group (1.83 [± 0.2] s). These findings show that the males in our pilot study had the same time duration to complete the STS movement as the participants in the two studies mentioned.

Our study showed the comparison between the perceived effort options, with the most common choice selected on the MBS being the ‘somewhat severe’ (33.33% [*n* = 3]) followed by the ‘severe’ (22.22% [*n* = 2]) category. However, when comparing the values between males and females, the male median rating was 4 (2.5 – 5.5), and the females was 3 (3 – 4). These values compared to the time taken are inversely proportionate, where the males completed the STS activity quicker (3.4 s [2.7–6.21]) compared to the females (6.08 s [5.5–7]). Yet the females rated the activity as ‘somewhat severe’ compared to the ‘severe’ rating of the males. The perception of STS will impact mobility at rehabilitation or home as it is a precursor to most mobility activities (Vena et al. 2015).

The association between EE and perceived effort during STS revealed a fair positive correlation between METs and MBS (*r* = 0.34); however, it did not reach statistical significance (*p*-value 0.38). Our pilot study aimed to determine the feasibility of a larger study and the fair correlation indicates that further research can be carried out with a larger sample size. Two other studies were also carried out to establish whether a correlation between perceived exertion and effort intensity existed. One reported that there was little to no correlation, *r* = 0.12 (*p* = 0.25) and the other showed a correlation between perceived exertion rating and measured effort intensity was poor (*r* = -0.04, *p* = 0.78) (Compagnat et al. 2017; Lacroix et al. 2019).

## Conclusion

The ability to come from STS is still seen as one of the most frequent activities used and the ability to complete it is the prerequisite to walking. Study participants’ exercise intensity during STS was much higher when compared to published literature in healthy individuals indicating the need to monitor exercise intensity levels during the rehabilitation process. The use of the MBS, during clinical practice, to monitor a patient’s effort level as a precursor for exercise intensity is thus a low-cost option. Although not statistically significant in our pilot study, a fair positive correlation between MBS and METs for patients with stroke during STS indicates that further research in this area is feasible. Establishing the EE in patients with stroke during STS could be a key point for rehabilitation.
